# Why Was Silcrete Heat-Treated in the Middle Stone Age? An Early Transformative Technology in the Context of Raw Material Use at Mertenhof Rock Shelter, South Africa

**DOI:** 10.1371/journal.pone.0149243

**Published:** 2016-02-11

**Authors:** Patrick Schmidt, Alex Mackay

**Affiliations:** 1 Department of Prehistory and Quaternary Ecology, Eberhard Karls University of Tübingen, Tübingen, Germany; 2 Centre for Archaeological Science, School of Earth and Environmental Sciences, University of Wollongong, Wollongong, Australia; 3 Department of Archaeology, University of Cape Town, Rondebosch, South Africa; Universidade do Algarve, PORTUGAL

## Abstract

People heat treated silcrete during the Middle Stone Age (MSA) in southern Africa but the spatial and temporal variability of this practice remains poorly documented. This paucity of data in turn makes it difficult to interrogate the motive factors underlying the application of this technique. In this paper we present data on heat treatment of silcrete through the Howiesons Poort and post-Howiesons Poort of the rock shelter site Mertenhof, located in the Western Cape of South Africa. In contrast to other sites where heat treatment has been documented, distance to rock source at Mertenhof can be reasonably well estimated, and the site is known to contain high proportions of a diversity of fine grained rocks including silcrete, hornfels and chert at various points through the sequence. Our results suggest the prevalence of heat treatment is variable through the sequence but that it is largely unaffected by the relative abundance of silcrete prevalence. Instead there is a strong inverse correlation between frequency of heat treatment in silcrete and prevalence of chert in the assemblage, and a generally positive correlation with the proportion of locally available rock. While it is difficult to separate individual factors we suggest that, at Mertenhof at least, heat treatment may have been used to improve the fracture properties of silcrete at times when other finer grained rocks were less readily available. As such, heat treatment appears to have been a component of the MSA behavioural repertoire that was flexibly deployed in ways sensitive to other elements of technological organisation.

## Introduction

Heat treatment of stone for knapping was one of the first fire-based transformative technologies used to alter the mechanical properties of natural materials. It was practiced from at least the second half of the Middle Stone Age (MSA) in southern Africa [1]. MSA heat treatment is known to have been applied to silcrete, a pedogenic silica rock [2] available along South Africa’s west and south coasts [3]. When silcrete is heated, it undergoes several physical and chemical transformations. From 250°C upward, chemically bound ‘water’ (SiOH) is lost from the structure, allowing for the formation of new Si-O-Si bonds [4] that transform the mechanical properties of the rocks [5]. These modifications of the material properties produce a tool-stone that can be worked more easily, as a result of various altered fracture properties including decreased fracture toughness [6, 7]. After heat treatment, the fracture behaviour of silcrete becomes closer to the one of finer grained silica rocks like chert and flint.

While heat treatment has been documented at several southern African sites, few data are available concerning its prevalence or the extent of variation within assemblages. At Pinnacle Point, on South Africa’s south coast, the majority of the silcrete from between 71 and 60 ka was heated [1]. This time interval corresponds with the production of microlithic technologies at the site which are similar to Howiesons Poort (HP) occurrences elsewhere. During the two so far analysed occupation phases in the HP at Diepkloof Rock Shelter, on South Africa’s west coast, heat treatment was a ubiquitous technique, applied to almost all silcrete before knapping [8]. At Blombos Cave, heat treatment was applied to some of the bifacial points from the Stillbay (SB) phase, dating ~74–71 ka [9], to prepare them for final retouch with a pressure flaking technique [10]. Documentation of silcrete heat treatment in the HP and SB reflects the fact that both of these periods are unusually silcrete-rich in the context of the broader MSA; in most sites through most of the MSA outside of the HP and SB silcrete is a marginal assemblage component which has received less archaeological attention.

Given the present paucity of data, factors governing the application of heat treatment and its temporal and spatial patterning remain relatively unknown. It has been argued that heat treatment was a necessary precondition for the use of silcrete, and that changing acquisition costs of fuel for heat treatment may have influenced the through-time prevalence of this rock type in archaeological assemblages [11]. While to an extent consistent with the HP data from Pinnacle Point and Diepkloof where high proportions of silcrete and high rates of heat treatment coincide, this hypothesis is difficult to reconcile with more selective application of heat treatment in the SB at Blombos. In order to approach some of the relevant controlling factors, in this paper we focus on the relationship between heat treatment of silcrete and other elements of assemblage variability in the southern African MSA. We consider three questions:

- *How consistent is the relationship between the prevalence of heat treatment and the relative proportion of silcrete in an assemblage*? Arguments suggesting that heat treatment was necessary for the use of silcrete would imply a fairly stable relationship between these two factors, with consistently high levels of heat treatment assumed to be associated with an abundance of silcrete.
- *To what extent does the presence of other fine-grained silica rocks influence the use of heat treatment*? If one of the effects of heat treatment is to improve silcrete’s fracture properties, the need for heat treatment may be offset by greater availability of finer-grained silica rocks such as chert.
- *Is heat treatment influenced by different patterns of landuse*? Allowing that variable mobility and provisioning through the MSA had the potential to constrain access to and transport of different rock types [12, 13], heat treatment may have provided a mechanism for foragers to increase yield from provisioned rocks at times of altered lithic resource availability.

**Fig 1:**
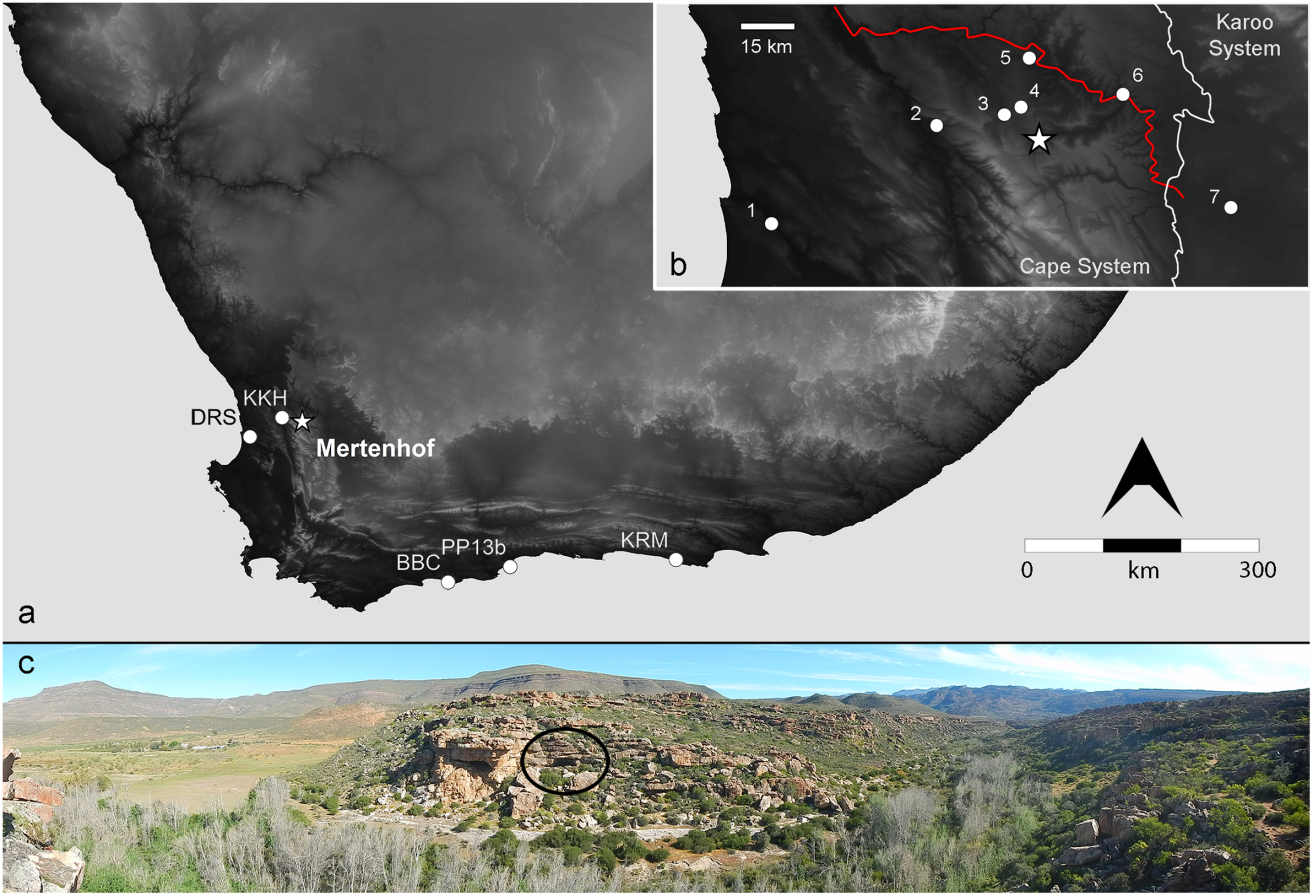
Overview and position of Mertenhof shelter. (a): Elevation model of southern Africa showing location of Mertenhof Rock Shelter (star) and other sites mentioned in the text (circles); BBC = Blombos Cave, DRS = Diepkloof Rock Shelter, KKH = Klein Kliphuis, KRM = Klasies River, PP13b = Pinnacle Point 13b. (b): Inset showing location of Mertenhof (star) relative to the Doring River (red line) and the boundary between the Cape System and Karoo System geological zones (white line). Other sites shown are 1 = Diepkloof, 2 = Klein Kliphuis, 3 = Hollow Rock Shelter, 4 = Klipfonteinrand, 5 = Putslaagte 8, 6 = Uitspankraal 7, 7 = Tweefontein. Digital elevation data from [21] (c): Lower panel shows a panorama of Mertenhof Rock Shelter (black circle) in the context of the kloof. The Ceberberg mountains are visible in the background on the right hand side of the image, and the shale-dominant Tra-traberge on the left.

**Fig 2:**
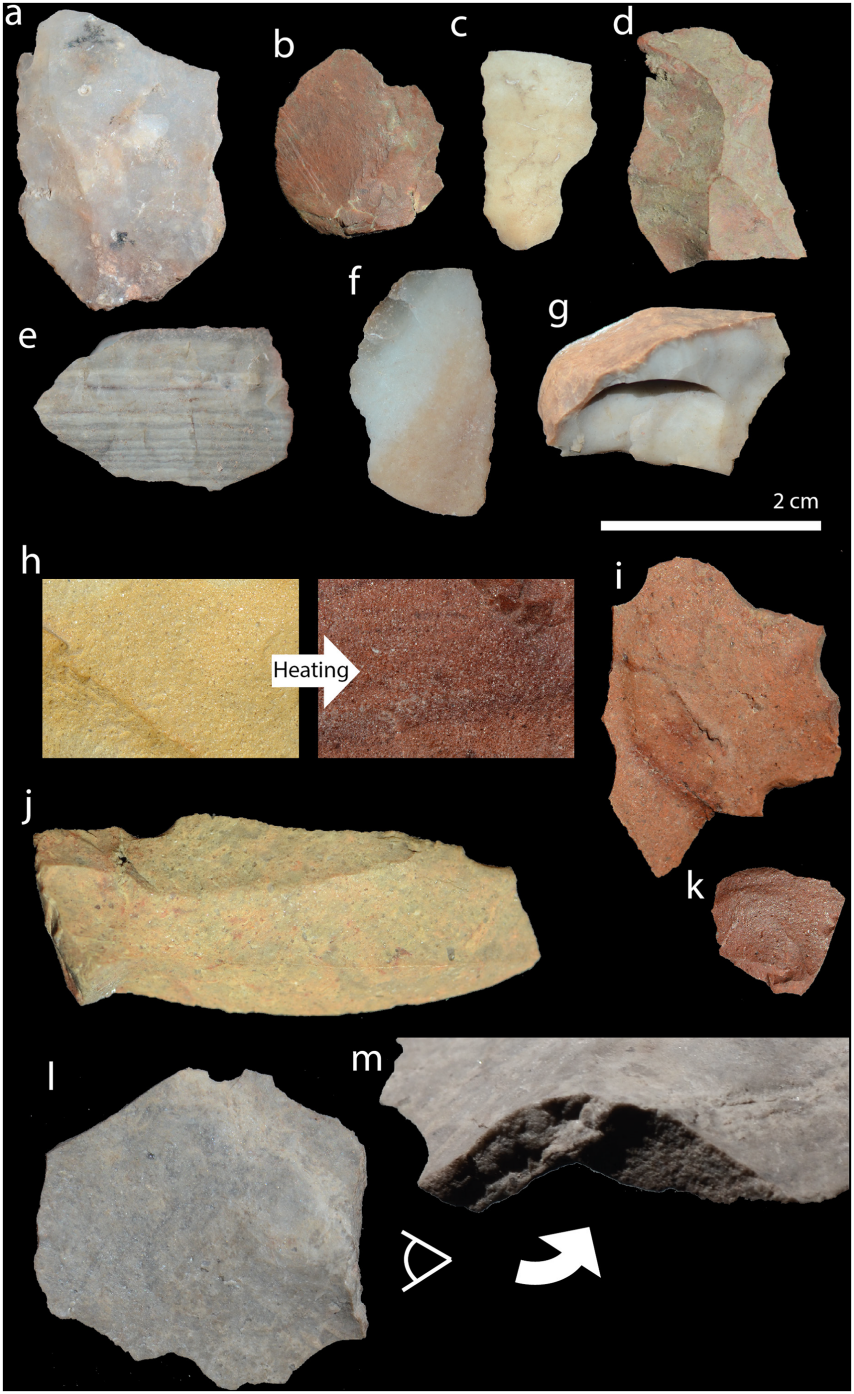
Chert and heat-treated silcrete artefacts in Mertenhof. (a-g): Different types of chert in the analysed Mertenhof assemblages. (h): Surface of unaltered and experimentally heat-treated silcrete showing the colour change upon heating. (j): Untransformed Mertenhof silcrete of the yellow type to which no heat treatment was applied. (i, k): Artefacts made from this same silcrete type that were heat-treated. (l): Mertenhof artefact made from grey silcrete. (m): the contrast between a pre- and a post-heating scar on the same artefact showing the difference of roughness. (a-g) and (i-l) all have the same scale. Note that the roughness of the surfaces in artefacts (i-l) cannot be estimated in these photos that were taken with frontal light.

To shed light on these issues, we investigated assemblages from a recently excavated site in South Africa’s eastern Cederberg region, Mertenhof Rock Shelter (MRS) (Fig 1). The site presents a reasonably long cultural sequence through the MSA, featuring multiple technological systems, and contains a significant component of silcrete [14]. Unusually for the region, the MRS assemblage is composed of a number of rock types with reasonably well-known and quite variable distances to source, allowing some understanding of changes in lithic transport to be developed. The assemblage includes up to 15% of fine grained silica rocks (henceforth called ‘chert’–Fig 2) in some stratigraphic units [14]; at other comparable regional sites peak proportions of chert rarely exceed 5% and silcrete is the only common ‘fine-grained’, predictably-fracturing rock [15–20]. MRS is therefore a useful location for exploring the questions of interest here.

## Methods and Materials

MRS is located in a narrow canyon of the Biedouw River dominated by quartzitic sandstones of the Table Mountain Series, close to the point where the river emerges into the more open valley formed in Bokkeveld Series shales and sandstones (Fig 1). Though a relatively small catchment the Biedouw provides running water year round in an area that is characteristically semi-arid, albeit that the river’s flow is greatly diminished in the hot, dry summer months. The reliability of water at this location likely underpins the strong and relatively persistent occupational signal in the shelter [22].

Of the main rock types in the MRS assemblage—quartzite, hornfels, silcrete, hydrothermal vein-quartz and chert—only quartzite and vein-quartz are known to be available within 20 km of the site. Quartzite of varying quality is ubiquitous throughout the region, occurring as massive bands including those of which MRS itself is formed. Vein-quartz (henceforth called ‘quartz’ for simplicity) occurs in the vicinity of the site as small pebbles eroding out of conglomerate beds, though the distribution of these is variable and package size is heavily constrained (generally <50 mm). Hornfels is available as cobbles exceeding 100 mm maximum dimension in the Doring River, 20 km NE, but does not appear to occur west of the river, and, in any case, most of the cortex on hornfels in the MRS is of cobble form. Primary silcrete has been identified on ridges 25 km to the SE (Agtesfontein) and 25 km to the NE (Swartvlei), but closer sources cannot be precluded. Secondary silcrete cobbles occasionally occur in the Doring River but these are generally coarser-grained than those located in primary contexts and appear to have seen limited use [23]. Chert can also be found as pebbles in the cobble beds of the Doring River (at ~20 km from site) but it is rare [23]; more predictable sources of chert occur in the Dwyka and Ecca Series formations of the Karoo System starting 35 km and 45 km east respectively, towards the increasingly arid interior [24].

MRS has so far been excavated over four seasons from 2013–2015, revealing late Holocene, Pleistocene Later Stone Age, late MSA, post-HP, HP, Still Bay and early MSA components [14]. The research permit to conduct archaeological excavations at Mertenhof is issued under the National Heritage Resources Act (Act 25 of 1999) and the Western Cape Provincial Gazette 6061, Notice 298 of 2003 and valid from April 2013–2016. AM is the permit holder (permit number: 130306TS13). All recovered lithic artefacts are temporarily housed in the Department of Archaeology at the University of Cape Town pending accessioning at Iziko South Africa Museum, 25 Queen Victoria Street, Cape Town, 8001, South Africa, where they will be available for further analysis. Mertenhof specimen numbers range from 1–12923 (season 1), 20000–29429 (season 2) and 30000–42600 (season 3). Heritage Western Cape (HWC) requires permits only for destruction of archaeological material. For the present study, no destructive analyses were performed and no additional permits were required.

Excavation was undertaken in a series of contexts, referring in this sense to a stratigraphically coherent aggregate. Most often these comprise small, distinct sedimentary units, though where sedimentary units exceed 30 mm in depth ‘context’ is used to refer to subdivisions of the unit (effectively spits following the last known slope).

We analysed a series of contexts (n = 7) through the major stratigraphic unit BGG/WS, covering the start of the HP through to the early phases of the post-HP. It is in this part of the sequence that silcrete is most abundant, generally exceeding 25% of artefacts (Table 1). To improve the robusticity of results for our analysis we only examined contexts with samples of more than 100 artefacts in total. Most of the contexts we analysed derive from excavation square 3, but to increase our sample of post-HP data we included one context (790) from square 4 (We have so far only undertaken basic classification of finds from MRS; these artefacts have not yet been subject to technological analysis).

**Table 1:** Raw material prevalence and implement types in the lithic sequence at MRS.

Context	Industry	n artefacts	% quartzite	% silcrete	% chert	% hornfels (+ DWS)	% quartz	n backed	n notch	n cores	Cores / 100 flakes
790	Post-HP	272	68.8	29.9	1.1	3.3	1.1	0	0	1	0.3
303	Post-HP	107	55.1	26.5	1.9	10.3	2.8				
306	HP (upper)	131	40.5	32.8	4.6	15.3	2.3	8	1	6	2.1
314	HP (upper)	225	20.4	43.1	8.4	16.9	4.9				
320	HP (middle)	111	21.6	20.7	9.0	30.6	3.6	10	4	18	4.8
321	HP (middle)	396	10.9	30.1	12.4	33.6	6.6				
328	HP (lower)	166	12	16.3	13.3	33.1	20.5	6	1	10	8.5

Context numbers are arbitrary and have been arranged in the table in stratigraphic order. Raw material %’s include only the most common rock types. DWS refers to degraded white stone. Values under ‘n backed’, ‘n notch’, ‘n core’ and ‘Cores / 100 flakes’ refer to all contexts belonging to the same industry.

As with other sites in the broader region, the basic characteristics of the HP at MRS vary through the sequence, including changes in raw material prevalence and in the abundance of backed and notched pieces [15, 17, 25] (Table 1). To characterize these changes, and to correlate them with the patterns of heat treatment-related behaviours observable on silcrete, we assessed the frequencies of all raw material classes in the analysed context. Changes in core to flake ratio and artefact density are also considered as a proxy for mode of technological delivery, or provisioning systems [26].

Heating patterns were evaluated as follows: For all contexts we analysed the silcrete artefacts for the presence/absence of heat treatment proxies according to the method described in Schmidt et al., [8]. This method is based on the principle that fracture surfaces which result from knapping after heat treatment (post-heating removal scars) have a noticeably lower surface roughness than fracture surfaces which developed in the unaltered silcrete (pre-heating removal scars). In order to recognise pre- and post-heating scars, the artefacts’ fracture surfaces are compared with an experimental reference collection of untreated and heat-treated silcrete. This makes it possible to assign silcrete artefacts to four categories: (1) artefacts with pre-heating removal scars only, i.e. not heat-treated artefacts; (2) artefacts with post-heating scars, i.e. heat-treated artefacts; a part of which are (3) artefacts with both, pre- and post-heating scars, i.e. heat-treated artefacts that document a stage of knapping or preforming prior to the heat treatment and (4) artefacts with surfaces due to heat-induced non-conchoidal (HINC) fracturing, i.e. that exploded in a fire. To avoid having artefacts that burned during post-depositional processes enter the category HINC, an artefact is assigned to this category only if the succession of removal scars leaves no doubt that knapping continued after the heat-induced fracturing, i.e. one or more post-heating scars cross-cuts the HINC fracture surface.

Compiling a meaningful reference collection for the Mertenhof assemblages is not straightforward because there is no silcrete source in the direct vicinity of the site. The closest known outcrops yield weathered blocks of yellow, sometimes grey silcrete that range from very coarse-grained to rather fine-grained. Larger pieces have knappable material below the brown weathering rind. We heat-treated a few blocks from these outcrops in a fire in order to produce a reference collection. The grey samples did not change colour but the yellow samples turned red (Fig 2h). Both types had noticeably smoother fracture surfaces after heat treatment. Because this reference collection may be incomplete, we refined our determination of pre- and post-heating scars through an ‘internal calibration’. For the yellow silcrete, this works as follows: First, some bright yellow artefacts are selected (cf. Fig 2j). This is the reference collection of unaltered silcrete (the degree of confidence is very good because heat treatment would have caused them to turn red). The roughness of the removal scars on these not heated artefacts is then compared with removal scars on red artefacts. Red artefacts with scars that are smoother than the scars on yellow artefacts were assigned to the category of heat-treated silcrete (cf. Fig 2i and 2k). Red artefacts with scars of similar roughness as the reference collection were identified as burned after discard but not heat-treated. Most of the Mertenhof artefacts were made from such yellow silcrete. A second component of the Mertenhof silcrete is the grey type that does not change colour upon heating (Fig 2l). The ‘internal calibration’ for this type was as follows: First, artefacts that show a clearly distinguishable roughness contrast between adjacent removal scars were selected (Fig 2m). Because the difference in roughness between two scars on the same artefact is produced by knapping before and after heat treatment (i.e. category (3)), these rough and smooth fractures were used to calibrate our identification of pre- and post-heating scars on the other artefacts made from this silcrete type. Mertehof artefacts that could not be identified as either of these two silcrete types were left undetermined. These undetermined silcrete artefacts were excluded from the study of heat treatment proxies and only included in the total count of silcrete in each context (this was the case of 9% of the silcrete from the analysed CTs). HINC surfaces were determined through the presence of concave angular structures and scalar features [8].

## Results

### Raw material frequencies and provisioning changes at MRS

Two of the characteristics setting MRS apart from most other MSA sites that lie in the Cape coastal zone are the relative degree of control available over raw material source locations (see introduction) and the abundance of chert in some of its assemblages. Quartzite is the only raw material available locally and predictably; hornfels has a minimum transport distance of 20 km, while silcrete may also be sourced from this distance. Only chert is likely to derive from east of the Doring River at a distance probably exceeding 35 km.

Four varieties of chert can be identified at MRS. Red radiolarite is recognisable by its typical markers of tectonisation and the inclusion of radiolarian micro-fossils (Fig 2b and 2d). The second type is a white structureless homogenous silica rock, most likely formed by chalcedony (Fig 2c). The third type is bluish transparent (most likely chalcedony) with brown inclusions and sometimes manganese dendrites (Fig 2a, 2f and 2g) and the last type is a blue opaque, finely bedded, silica rock (Fig 2e).

To evaluate the quantity of hornfels in the MRS assemblages, another material class must be taken into account, what we refer to as DWS (degraded white stone). DWS exhibits an inverse proportional distribution to hornfels through the sequence [22], and is entirely absent from the uppermost (Holocene) strata in the site. The extent of decay increases through the sequence such that upper examples are greyish-brown and generally very similar to hornfels, while those towards the base are white and quite powdery, regularly fragmenting on recovery. That there are visually intermediate stages in this decay sequence, combined with the hornfels-like nature of the upper examples and the inverse relationship between proportions of the two leads us to conclude that the one rock (hornfels) is decaying into the other (DWS). Although the processes leading to this selective alteration process need further study, we combine the count of DWS and hornfels for this study to obtain a meaningful percentage of hornfels in the assemblages.

Frequencies of all raw materials in the analysed contexts are summarized in Table 1. In the lowest HP, silcrete is proportionally less common but values for quartz and chert are elevated. In the middle HP contexts, chert declines while silcrete becomes more common and in the uppermost HP, silcrete reaches its highest proportions. Quartzite increases from the bottom of the HP through to the post-HP, becoming the dominant rock type in that grouping, though silcrete remains prevalent.

Changes in raw material prevalence are to some extent matched by changes in provisioning, assessed here in terms of the relative frequencies of cores. In the lowest HP cores are proportionally abundant, occurring at the rate of 8.5 per hundred flakes (or one core per 11.7 flakes). Core proportions decline through the sequence reaching a minimum value in the post-HP. The two post-HP contexts analysed contained only one core between them, and a combined total of 341 flakes.

### Heat treatment of silcrete at MRS

Heat treatment proxies were observed on silcrete artefacts from all HP and post-HP contexts examined at MRS. This included evidence for flaking post thermal fracture (HINC) and also tentative evidence for artefacts with tempering residues following Schmidt et al. [8].

Frequencies of heat treatment proxies are summarised by context in Table 2 and by industrial (sub)grouping in Table 3. The prevalence of heat treatment is highly variable overall, ranging between 88.7% and 36.5% of silcrete artefacts by context, and between 86.9% and 36.8% by industrial grouping. Heat treatment is least common during the lower HP and most common in the post-HP, occurring in the latter at frequencies comparable to those observed in the two HP layers analysed at Diepkloof [8]. The percentage of silcrete artefacts that document a stage of knapping or preforming prior to heat treatment is less variable, though flakes with pre-heat treatment surfaces are most common in the middle and upper HP layers. The frequencies of HINC fractures are low overall and absent in the silcrete-poor lower HP, but otherwise show a sequential decline from the middle HP to the post-HP.

**Table 2:** Frequency of heat treatment proxies on the silcrete artefacts for all contexts.

Context	Industry	Heat-treated (%)	Pre-HT surface (%)	HINC (%)
790	Post-HP	88.7	12.8	4.3
303	Post-HP	85	35.3	0
306	HP (upper)	77.1	22.2	7.4
314	HP (upper)	48.8	20	0
320	HP (middle)	50	11.1	11.1
321	HP (middle)	36.4	25	6.3
328	HP (lower)	36.8	14.3	0

Heat treated (%) refers to proportion of silcrete artefacts with signs of heat treatment in terms of the criteria outlined above and in [8]. Percentages under ‘% heat-treated silcrete’ are relative to the total number of silcrete artefacts. Percentages under ‘Pre-HT surface (%)’ and under ‘HINC (%)’ are relative to the total number of heat-treated artefacts.

**Table 3:** Frequency of heat treatment proxies on the silcrete artefacts by industrial (sub)grouping relative to raw material proportions for main rock types.

Industry	% silcrete	% chert	% quartzite	% hornfels + DWS	Heat-treated (%)	Pre-HT surface (%)	HINC (%)
Post-HP	27.4	1.3	64.9	5.3	86.9	18.8	3.1
HP (upper)	39.3	7	27.8	16.3	63	21.3	4.3
HP (middle)	28	11.6	13.2	32.9	43.2	22	7.3
HP (lower)	16.3	13.3	12.1	33.1	36.8	14.3	0

Heat treatment criteria as per Table 2.

### Correlation of raw material frequencies, provisioning and heating treatment

With respect to the questions posed at the start of this paper we can consider the relationships between heat treatment and the prevalence of different rock types by using both individual contexts and with respect to our industrial groupings and subgroupings. The latter approach provides more robust overall sample while the former obviates the assumption of validity in our industrial subgroupings.

First, we consider whether there is a relationship between the prevalence of silcrete in the assemblage and the frequency with which it is heated. At both the context and industrial (sub)grouping level there is no clear or statistically significant relationship between the relative abundance of silcrete and the frequency with which it was heated (by context: r^2^ = 0.036, p = 0.682; by (sub)grouping: r^2^ = 0.203, p = 0.549) (Pearson’s r = 0.375, p = 0.204 and r = 0.398, p = 0.301 respectively).

**Fig 3:**
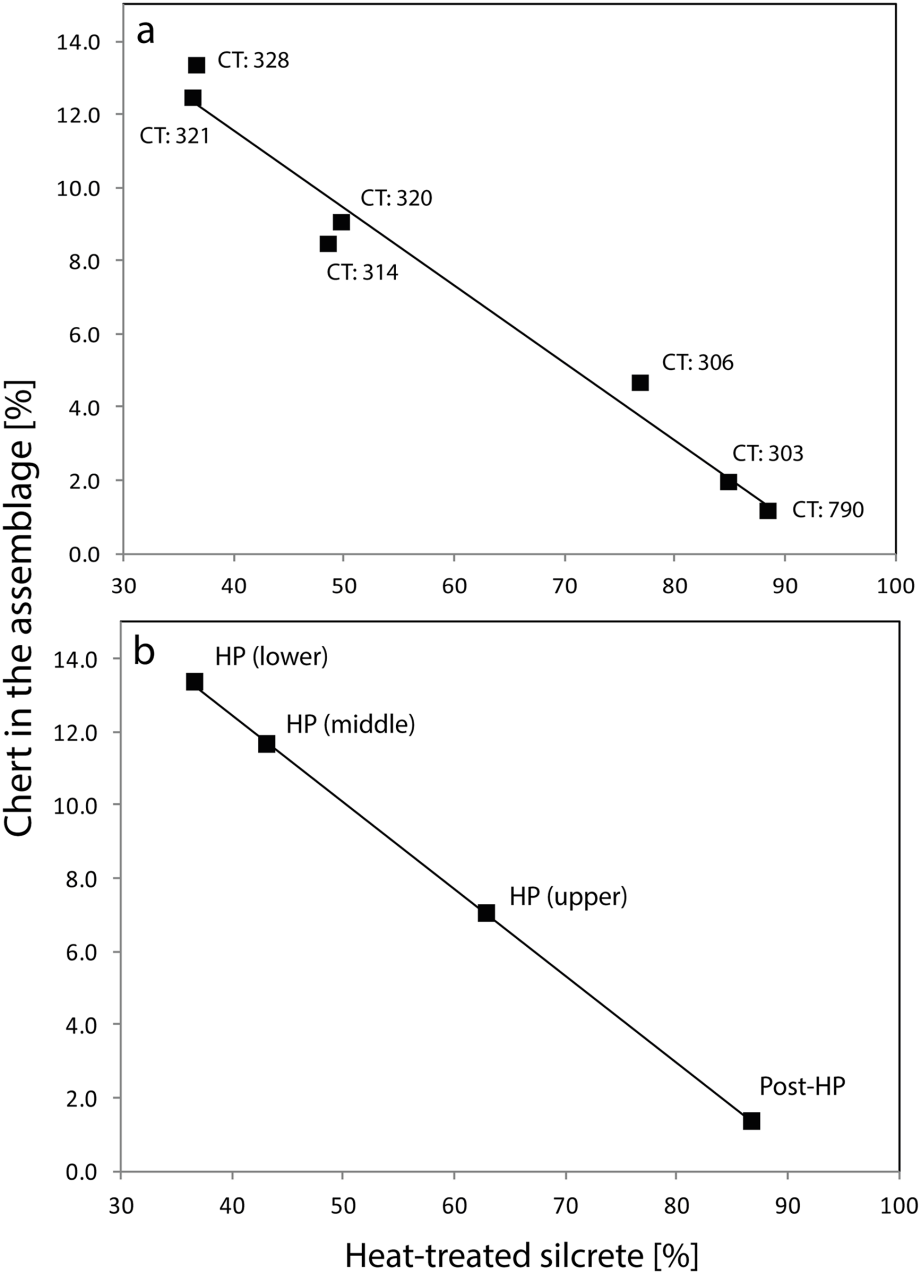
Correlation plots showing the relation of the percentage of chert artefacts in relation to the percentage of heat-treated silcrete. (a) By contexts (CT) and (b) by (sub)groupings. Chert percentages refer to the total number of artefacts in the contexts, heat-treated silcrete percentages refer to the number of silcrete artefacts in the contexts. The coefficient of determination of the correlation is r^2^ = 0.973, p<0.001 for (a) and r^2^ = 0.999, p<0.001 for (b).

The second question we posed was whether there was a relationship between the prevalence of heat treatment and the frequency of non-silcrete fine-grained rocks such as chert in the assemblage (Fig 3a and 3b). The underlying concept was that if heat treatment of silcrete operated to make its fracture characteristics more chert-like, then the prevalence of chert may have limited this need. Consistent with this suggestion, there is a clear inverse and statistically significant relationship between the prevalence of chert and frequency of heat treatment in both the context-level (r^2^ = 0.973, p<0.001) and (sub)grouping data (r^2^ = 0.999, p<0.001) (Pearson’s r = -0.987, p<0.001 and r = -0.999, p<0.001 respectively).

**Fig 4:**
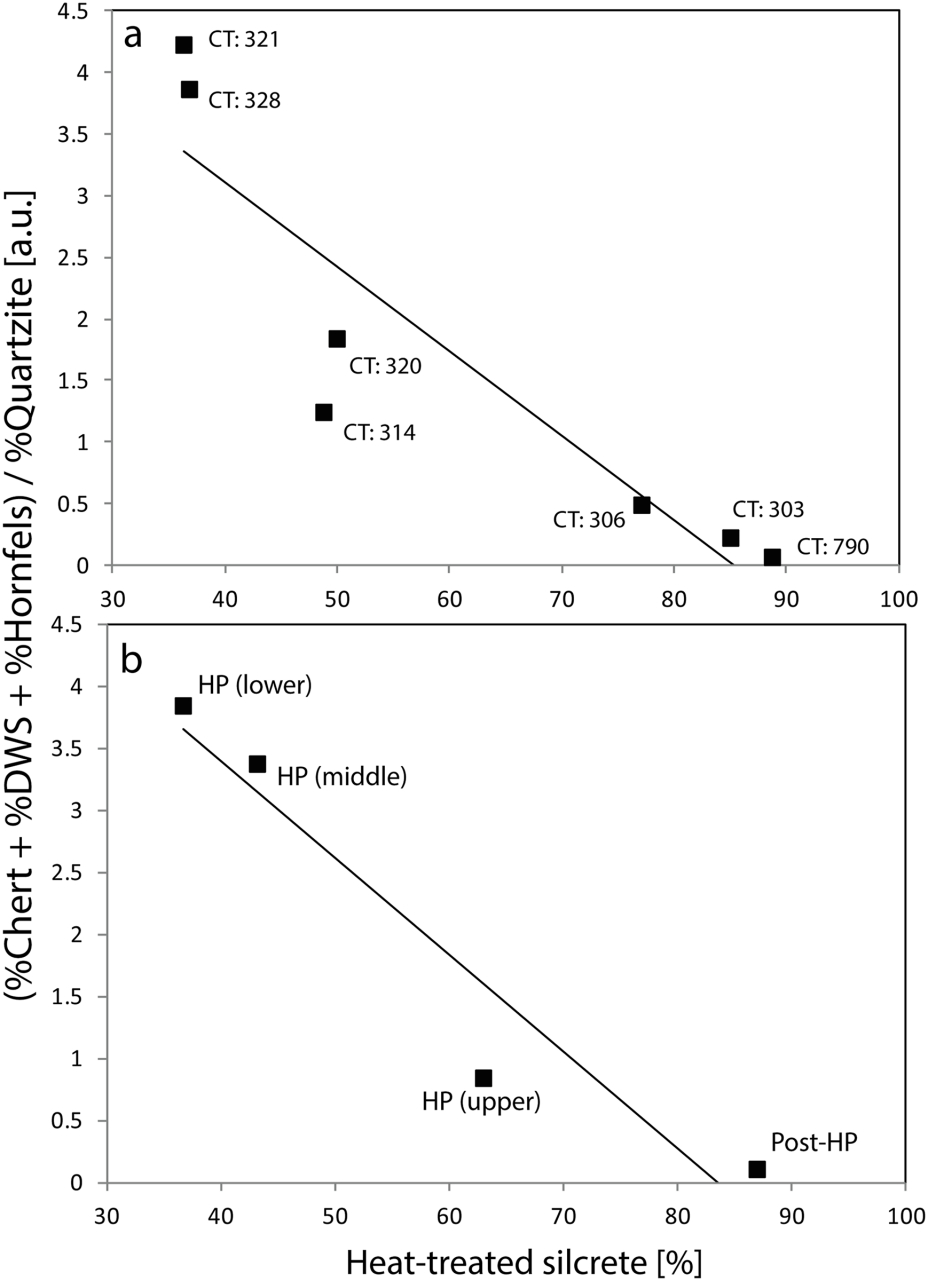
Correlation plots showing the relation of the raw material spectrum’s shift from fine-grained non-local materials towards coarse-grained local quartzite to the percentage of heat-treated silcrete. (a) By contexts (CT) and (b) by (sub)groupings. The non-local fine-grained / local coarse-grained materials ratio is calculated as the addition of the percentages of chert and DWS/hornfels divided by the percentage of quartzite in each context. Heat-treated silcrete percentages refer to the number of silcrete artefacts in the contexts. The coefficient of determination of the correlation is r^2^ = 0.823, p = 0.005 for (a) and r^2^ = 0.921, p = 0.040 for (b).

**Fig 5:**
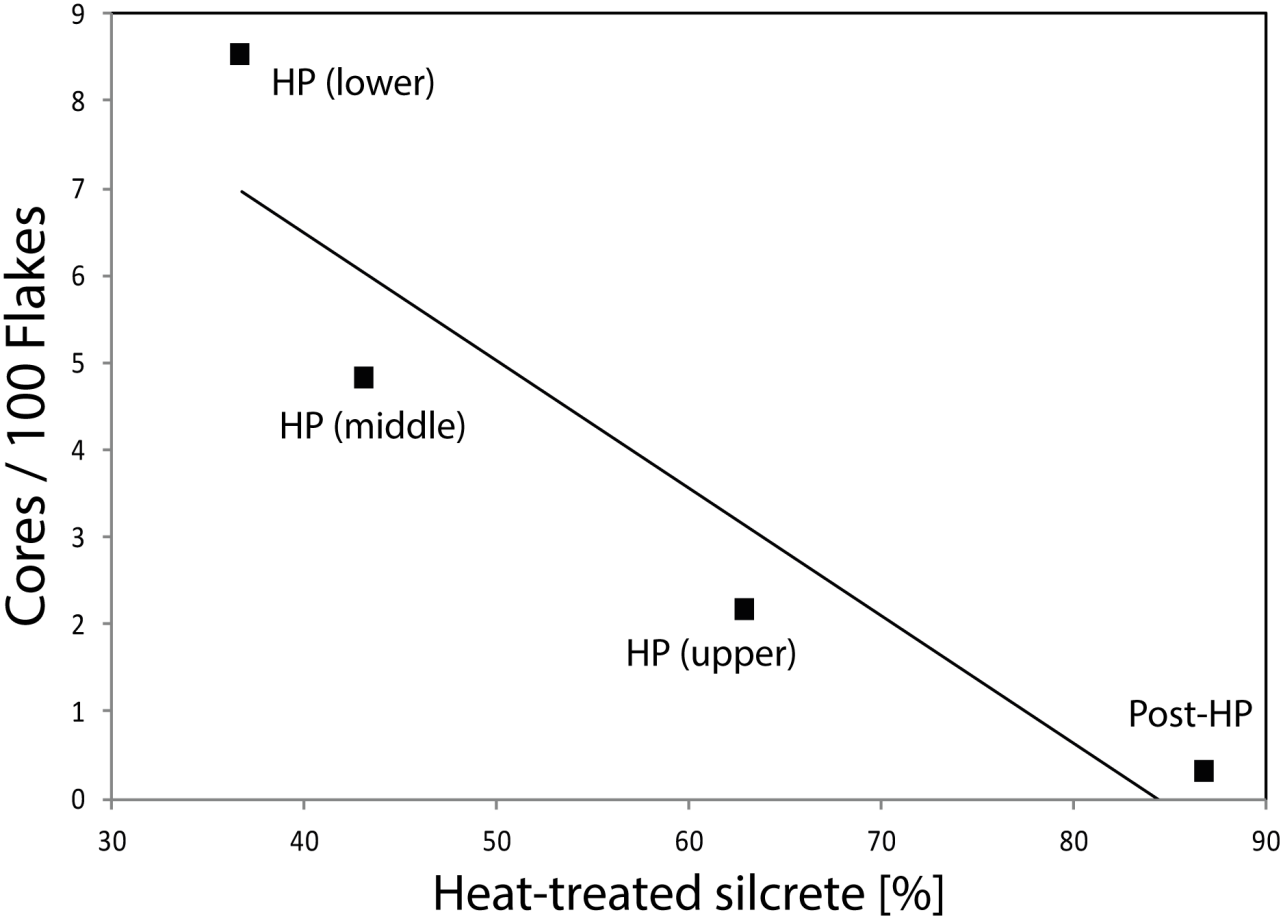
Correlation plots showing the relation between the number of cores/flakes and heat-treated silcrete in (sub)groupings. Cores/100 flakes are calculated from the total number of artefacts in the (sub)groupings and heat-treated silcrete percentages refer to the number of silcrete artefacts in the contexts. The coefficient of determination of the correlation is r^2^ = 0.859, p = 0.073.

Interpretation of the relationship between heat treatment and prevalence of chert has the potential to be confounded by changes in mobility. That is, heat treatment may be varying in response to changes in chert, or it may be that heat treatment is responsive to general changes in the acquisition of non-local rocks, of which chert is one. Examination of variation in heat treatment of silcrete relative to the ratio of non-local rocks chert and hornfels/DWS to the locally abundant coarse-grained quartzite (Fig 4) implies that this latter proposition may have merit. Even in the unlikely event that DWS is not hornfels, where visible, the cortex on this rock reflects fluvial transport, with the Doring or some more distant river as its most likely source (the cobble bedload of the Biedouw comprises sandstone, quartzite and quartz). There is a weak negative correlation between the prevalence of silcrete heat treatment and the relative abundance of all non-local fine-grained rocks in both the context-level (r^2^ = 0.823; p = 0.005) and (sub)grouping data (r^2^ = 0.921, p<0.040) (Pearson’s r = -0.902, p = 0.002 and r = -0.96, p = 0.02 respectively). Extending this observation, we note that the relative abundance of cores through the sequence is also weakly negatively correlated with heat treatment of silcrete (r^2^ = 0.859, p = 0.073; Pearson’s r = -0.927, p = 0.037) (Fig 5).

## Discussion

### The MRS pattern in the light of heat treatment in the MSA

The general prevalence of heat treatment at MRS stands in contrast to analysed HP assemblages at other sites such as Diepkloof and Pinnacle Point where the majority of silcrete appears to have been heated [1, 8]. At MRS, heat treatment was significantly less common in the HP than during the post-HP. Only in the post-HP does the prevalence of heat treatment come close to the frequencies known from Diepkloof. This clearly demonstrates that the prevalence of silcrete heat treatment was not tightly associated with the HP techno-complex. It rather seems to have been driven by other factors (see below).

Our data also allow some observations of the techniques or procedures used to heat silcrete at MRS: HINC fractures on the material indicate that, like at Diepkloof, silcrete exploded in the fire during heat treatment. Although the frequency of HINC-fractures in the middle HP layers at MRS is comparable to the intermediate HP at Diepkloof, indicating a procedure that involved fast heating, the lower percentages in the other layers might point toward slightly variable modes of heating through time. A residue, macroscopically similar to tempering residue [8] could also be observed on some pieces from MRS but only future chemical analyses can confirm its nature. Even if the presence of tempering residue is confirmed, it seems from our sample to be far less common than in Diepkloof. Whether these differences in the number of HINC fractures and the rarity, or absence, of tempering residue are caused by the nature of the silcretes used, the specific floral environment in the eastern Cederberg or spatial and temporal variations in the heating technique cannot be decided on the basis of our results. These thoughts aside, given our data, it seems reasonable to state that the extent to which heat treatment was applied was spatially variable through the MSA.

### The acquisition of silcrete does not imply heat treatment of silcrete

We could not find support for the idea that the use of silcrete was contingent on the application of heat treatment, or that an increased use of silcrete would necessarily result in a greater frequency of heat treatment. In the HP layers at MRS, silcrete was often introduced to the site, flaked and discarded in an unheated state. The relationship between heat treatment and silcrete abundance is not simple or linear.

### Heat treatment of silcrete appears to be responsive to the availability of other fine grained rocks

Another potential factor in rates of heat treatment may be the relative prevalence of chert, assuming that heat treated silcrete is more chert-like in its knapping characteristics. As noted earlier, chert is not common at many Cape MSA sites, and MRS is an exception. In our analysed sample there was a clear inverse relationship between heat treatment of silcrete and proportion of chert, implying that an absence of the latter may have encouraged application of the former. While landuse effects may be confounding this relationship (see below), its plausibility is implied by the limited intermediate HP data we have available from units Frans and Frank at the site of Diepkloof (DRS). These two layers, which have very little chert (3.4% and 1.3% respectively; Mackay data), have abundant evidence for heat treatment of silcrete (93.7% and 96.7% respectively [8]). Addition of these data to those from MRS substantiates the observed pattern (heat treatment of silcrete vs % chert by context, DRS data included: r^2^ = 0.927, p<0.001; Pearson’s r = -0.97, p<0.001). The further test conditions for this assertion are clear: we expect sites with high rates of both chert and silcrete to show lower frequencies of heat treatment than those in which silcrete is common but chert is not.

### Heat treatment of silcrete is probably influenced by changing landuse and provisioning patterns

The frequency of chert is not the only factor showing a strong relationship with the frequency of silcrete heat treatment in MRS; landuse and raw material provisioning are also implicated. From the early HP to the post-HP the prevalence of the non-local rocks chert and hornfels undergo steady decline, with most of the shortfall being taken up by an increase in the only consistently available local rock, quartzite. At the same time, cores were discarded less often, potentially reflecting less transportation of tool-making potential to the site. That this change is caused by either restrictions of raw material access or changing tool transport systems is highlighted by raw material selection patterns from the selected post-HP sample at Tweefontein published by Hallinan and Shaw [27], located 52 km SE of MRS in chert-bearing Dwyka geological series, where chert accounts for ~13% of artefacts and hornfels for 45%. Thus, chert and hornfels were still favoured as raw materials in the region during the post-HP, but their transportation to MRS in this period was greatly diminished. That heat treatment of silcrete occurred in the context of dwindling supplies of fine-grained rocks from distant sources and increasing dependence on local rocks conceivably reflects attempts by knappers to improve the knapping qualities and increase the flaking yield obtained from acquired silcrete. In other words, heat treatment may have been a substitute for other ‘good quality’ raw materials when access to them was restricted.

## Time as a Potential Confounding Factor

All of our discussion so far has focussed on specific factors for which we could make predictive statements. Some of these statements were consistent with the data from MRS, and others were not. We also noted the potential for confounding effects between inter-related variables, such as the effects of chert and mobility on heat treatment. One factor which we have not explicitly considered is the effect of time. Specifically, it could be that heat treatment is subject to a form of cumulative evolution, such that its relative proportion increases through time independent of changes in other variables. Heat treatment would thus be subject to its own evolutionary trajectory. Under such a scenario, statistically-significant relationships between heat treatment and other factors would be rendered largely coincidental. Given its implied insensitivity to economic considerations, we do not consider this possibility especially likely, however its test conditions are clear: increases in proportions of heat treatment from the SB through the HP into the post-HP should be a general feature of silcrete-rich lithic assemblages sharing those technological characteristics.

## Conclusion

The data we have presented allow us to draw a number of conclusions about the application of heat treatment during the MSA in southern Africa. The simplest of these is the extension of the cultural/technological contexts within which heat treatment has been observed. Though previously demonstrated in the HP [8, 15] and SB [10], and perhaps also in the early MSA [1], heat treatment has not previously been documented in the post-HP. While a minor and unsurprising finding, this serves to reinforce earlier arguments [28] countermanding persistent characterisation of the post-HP as a time of diminished behavioural complexity. While some behaviours appear to have been lost at the end of the HP [29], many others transformed [30–32], and still others, such as the use of heat treatment, appear to have persisted.

More substantially, our study of the MRS assemblages strongly indicate that, despite its prevalence at other sites like Diepkloof and Pinnacle Point, heat treatment was not a blanket strategy employed by MSA people whenever silcrete was accessed. In some horizons many tools were knapped from untransformed not heat-treated silcrete, documenting that the production of MSA tools does not generally require heat treatment and that heat treatment is not necessary to make silcrete functional as was suggested previously [11]. Instead, heat treatment was spatially and temporally variable, and its deployment was sensitive to other elements of the technological system including raw material availability and potentially also patterns of landuse and provisioning. On this basis, we argue that heat treatment-related behaviours in the MSA may have, in part, had an economic basis, controlled by variable access to other fine-grained rocks, and particularly chert. To that extent the data from MRS reinforce the perception of MSA foragers as behaviourally subtle, flexible and innovative.
